# Development of a Methodology for Determination of Dioxins and Dioxin-like PCBs in Meconium by Gas Chromatography Coupled to High-Resolution Mass Spectrometry (GC-HRMS)

**DOI:** 10.3390/molecules28135006

**Published:** 2023-06-26

**Authors:** Iñaki Lacomba, Antonio López, Raquel Hervàs-Ayala, Clara Coscollà

**Affiliations:** 1 Foundation for the Promotion of Health and Biomedical Research of the Valencian Region, FISABIO-Public Health, Av. Catalunya, 21, 46020 Valencia, Spain; lacomba_ina@gva.es (I.L.); lopez_anttob@gva.es (A.L.); 2 Department of Analytical Chemistry, Univeristy of Valencia, Doctor Moliner 50, 46100 Burjassot, Spain; 3 Department of Obstetrics and Gynaecology, General University Hospital of Valencia, 46014 Valencia, Spain; hervas_raq@gva.es

**Keywords:** dioxins, dl-PCBs, meconium, human biomonitoring, high-resolution mass spectrometry

## Abstract

An analytical strategy was applied to investigate polychlorinated dibenzo-p-dioxins (PCDDs), polychlorinated dibenzofurans (PCDFs), and dioxin-like polychlorinated biphenyls (dl-PCBs) in newborn meconium samples. The methodology includes extraction by selective pressurized liquid extraction (SPLE), followed by a clean-up multicolumn step. The samples were injected by gas chromatography coupled to a high-resolution mass spectrometer (GC-HRMS). The surrogate recoveries ranged from 68% to 95%, and the average of the limit of quantification (LOQ) ranged from 0.03 to 0.08 pg g^−1^ wet weight (ww) for PCDD/Fs and 0.2 to 0.88 pg g^−1^ ww for dl-PCBs. The strategy was applied to 10 samples collected in Valencia (Spain) in 2022. In total, 18 out of 29 analysed congeners were detected in at least one sample, whereas 6 of them were detected in all the samples (OCDD, PCB-123, PCB-118, PCB-105, PCB-167, and PCB-156). The levels for the sum of the 17 congeners of PCDD/Fs and 12 congeners of dl-PCBs in the upper-bound (UB), expressed as picograms of toxic equivalency quantity (TEQ) per gram of ww, ranged from 0.19 to 0.31 pg TEQ g^−1^ ww.

## 1. Introduction

The terminology “dioxins and dioxin-like substances” commonly encompasses polychlorinated dibenzodioxins (PCDDs), polychlorinated dibenzofurans (PCDFs), and polychlorinated biphenyls (PCBs). These chemical entities are characterized by two-or three-ring molecular structures that exhibit varying degrees of chlorination. PCBs can exhibit up to 10 chlorine atoms substituting for hydrogen atoms, whereas PCDDs and PCDFs can possess up to 8 chlorine atoms. These compounds often demonstrate comparable toxicity profiles and share common mechanisms of action, thus warranting their collective consideration as a cohesive group when establishing regulatory guidelines [1]. Human exposure to dioxins (PCDDs and PCDFs) and PCBs is a problem of global concern due to the fact that they are unintentionally produced. PCDD/Fs and PCBs are toxic bioaccumulative organic compounds, and they have been classified as persistent organic pollutants (POPs) by the Stockholm Convention [2]. Certain types of PCBs share similarities in structure and toxicity with PCDD/Fs and are referred to as dioxin-like PCBs (dl-PCBs) [3]. There are 17 PCDD/Fs and 12 dl-PCBs that have been identified as harmful to human health, with a broad range of toxicity, including dermal toxicity and immunotoxicity and negative effects on reproduction, embryo development, and endocrine functions [4,5]. In newborns, these pollutants can cross the placental barrier and enter the fetal bloodstream [6]. Furthermore, prenatal exposure to dioxins can cause different adverse health effects such as Yusho disease, among others [7].

Human exposure to dioxins and dl-PCBs can be by ingestion, inhalation, and the dermal pathway. Due to their lipophilicity and low water solubility, PCDD/Fs and dl-PCBs are persistent compounds that can be found in the environment and accumulate in animal fat, being introduced into the food chain [8]. Dioxin exposure and its impact is a matter of concern because the population is exposed, mainly by ingestion, to these pollutants for long periods of their lives.

In order to study the internal exposure of dioxins and dl-PCBs, the most common matrices employed in human biomonitoring (HBM) are blood and breast milk due to their relative ease of collection and the presence of fats in both fluids [9]. Taking into account all the sources and routes of uptake typically considered in HBM studies, an exposure assessment to PCDD/Fs and dl-PCBs in lactating mothers and breastfed infants in the Valencian Region (Spain) was previously performed by our research group [10]. While, overall, the results were below the reference level established by the EFSA, it was noted that this level was exceeded in the case of the 95th percentile or maximum level [11].

Meconium is a less commonly used matrix in HBM studies. However, it has several advantages, for example, it is a non-invasive matrix, a large amount of sample can be obtained, and it contains information on long exposure [12]. The foetus can be exposed to a large number of chemicals; the majority of them tend to accumulate and deposit in the meconium. Meconium is an excellent depository for persistent compounds and provides a historical record of prenatal exposure to several environmental compounds [7]. Therefore, the assessment of dioxins in meconium is particularly suitable for monitoring the levels to which the foetus has been exposed during the last 3 months [13].

Taking into account the described literature, dioxin and dl-PCB extraction from meconium is not standardized, and it has been performed using several techniques, such as a vortex [14], an ultrasonic bath [15], Soxhlet extraction [16,17], or selective pressurized liquid extraction (SPLE) [2,18], with different solvents, such as hexane, dichloromethane, acetonitrile, and acetone and different mixtures of them.

Meconium has already been used in the past to study foetal exposure to other substances, such as pesticides belonging to different classes and their metabolites [19], volatile organic compounds (VOCs) [20], or heavy metals [21], using liquid chromatography coupled to a tandem mass spectrometer (LC-MS/MS). In order to measure PCDD, PCDF, and dl-PCBs in HBM studies, the use of gas chromatography coupled to a high-resolution mass spectrometer (GC-HRMS) is widely used to analyse other biological matrices, such as breast milk and blood [10,22].

To our knowledge, there is only one study in the literature that analyses dioxins and dl-PCBs together in meconium samples by GC-MS. However, this study does not analyse all the congeners described by the Stockholm Convention [7]. Other studies in the literature describe the analysis of dl-PCB congeners but none of them combines this with PCDD/Fs [2,17,18]. As far as we know, no work has been published related to the analysis of the 17 dioxin congeners and the 12 dl-PCBs cited in the Stockholm Convention in meconium samples by GC-HRMS.

The objective of this study was to develop an analytical strategy for analysing PCDD/Fs and dl-PCBs using HRMS instead of LRMS (low-resolution mass spectrometry) in order to better identify these compounds in a complex biological matrix. The methodology was applied to 10 samples of meconium collected from newly born children in the Valencian Region (Valencia, Spain).

## 2. Results and Discussion

### 2.1. Sample Preparation Procedure Results

To assess the different extraction methodologies, several parameters were compared, such as efficiency, reproducibility, and applicability. Overall efficiency was assessed by taking into account the sampling preparation time and surrogate recoveries. The reproducibility was assessed by considering each methodology and each congener within the methods. Finally, the applicability of each methodology was compared according to the properties of the matrix in question.

A key aspect to consider is the amount of extracted fat in each procedure, due to the fact that PCDD/Fs and dl-PCBs are lipophilic, and the results of these pollutants in HBM are usually expressed per g of fat. The concentration of these chemical compounds may widely vary depending on the amount of extracted fat in each extraction methodology, so this will be an important factor when deciding on the optimal method for extraction.

**Figure 1:**
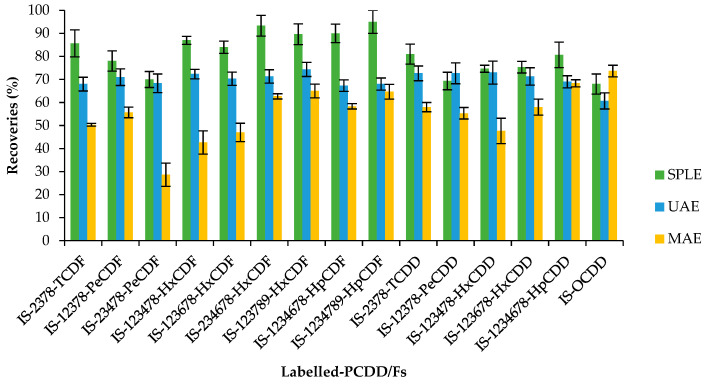
Recovery rates (%) and standard deviation of ^13^C_12_-PCDD/Fs (internal standards, IS) by selective pressurized liquid (SPLE), ultrasonic-assisted (UAE), and microwave-assisted (MAE) extractions. Each method was performed in triplicate.

**Figure 2:**
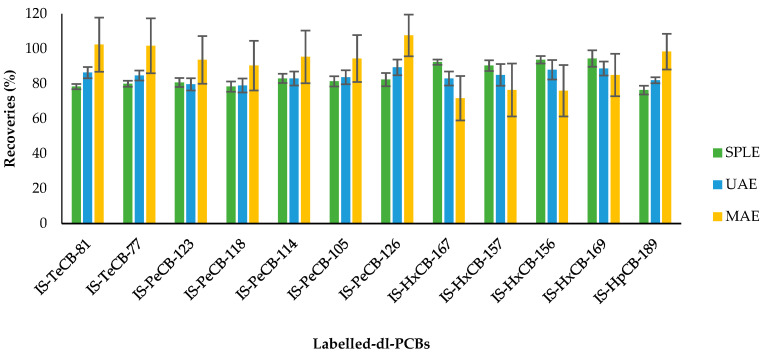
Recovery rates (%) and standard deviation of ^13^C_12_-PCBs (internal standards, IS) by selective pressurized liquid (SPLE), ultrasonic-assisted (UAE), and microwave-assisted (MAE) extractions. Each method was performed in triplicate.

Figure 1 and Figure 2 show the recoveries and standard deviations of the isotopically labelled congeners for the different studied groups.

For PCDD/Fs, the SPLE is the one that obtained the best results, obtaining an average recovery for surrogates of 81%, overcoming the obtained recoveries (except for 12378-PeCDD) in the other studied methodologies and obtaining good reproducibility values (RSD < 20%). The surrogate recoveries of UAE were very similar to those obtained with SPLE (average recovery of 70%), but it is important to highlight that by using the UAE methodology, ten times less fat was extracted. Surrogate recoveries were not appropriate (less than 60%) for several congeners using MAE extraction (average recovery of 56%), probably due to the fact that the matrix was calcined although toluene was employed as a solvent (boiling point 110 °C), ruling out this extraction methodology to analyse PCDD/Fs in meconium. MAE extraction was not previously employed to analyse POPs in meconium samples.

Regarding the obtained results for dl-PCBs using the different extraction techniques, the obtained recoveries for the labelled compounds are quite similar, and all of them meet the quality control requirements (Figure 2). As can be observed, the standard deviation of the surrogates using MAE was notably higher than the other two extraction techniques. The standard deviation for the dl-PCB surrogates was, overall, lower using SPLE than UAE.

After assessing these three extraction techniques and taking into account the obtained results for PCDDs, PCDFs, and dl-PCBs, SPLE is considered the most appropriate. The calculated LOQ for the PCDD/Fs ranged from 0.03 to 0.08 pg g^−1^ ww, and for dl-PCBs, the LOQ oscillated from 0.20 to 0.88 pg g^−1^ ww.

Some extraction methodologies and clean-up steps for the determination of these contaminants in meconium have been described in the Supplementary Materials (Table S1). In previous studies [16], the use of Soxhlet extraction, which has not been compared in this work, was used to analyse PCB-118 (one of the dl-PCBs evaluated in this study). Due to the large amount of time and solvent it consumes, more and more green chemistry methods are being developed in recent years. SPLE is a faster and more efficient extraction method, in addition to facilitating sample preparation and increasing throughput, as it allows combining the different steps of sample preparation.

### 2.2. Analysis of Real Samples

The results of PCDD/Fs and dl-PCBs are usually expressed by taking into account the amount of fat in the sample since they are lipophilic compounds. In this study, due to the characteristics of meconium, the levels are expressed in the wet weight of the sample. Taking the regulation of dioxins in food and feed as a reference, it says that the results must be expressed in fresh weight if the percentage of fat in the sample is less than 2%, as was the case for most of our samples [23].

Table 1 shows an overview of the PCDD/F levels by the congeners of the analysed samples in pg g^−1^ ww. The PCDD/F congener concentrations in the meconium ranged from not detected (n.d.) to 1.93 pg g^−1^ ww (OCDD). Regarding the detection frequencies (DF), 2,3,7,8-TCDF, 1,2,3,7,8-PeCDF, 1,2,3,4,6,7,8-HpCDD, and OCDD were by far the most detected, with 80, 90, 80, and 100% frequency detection, respectively. Regarding the other congeners, only two more (1,2,3,4,6,7,8-HpCDD and OCDD) were detected. Concerning the individual contribution of the PCDD/Fs to the total lower-bound (LB) pg TEQ g^−1^, 2,3,7,8-TCDF showed the major contribution, being almost 50% of the total contribution (48.8%), followed by 1,2,3,7,8-PeCDF (28.3%).

On the other hand, Table 2 shows the levels of dl-PCBs. In this case, all the congeners were detected in at least 20% of the analysed samples, whereas six of them were detected in all the analysed samples. The concentrations of dl-PCBs ranged from n.d. to 37.42 pg g^−1^ ww (PCB-118), with PCB-118 (32.1%) and PCB-169 (17.1%) contributing the most to the total LB.

To the best of our knowledge, this study is the first one to express the results of PCDD/Fs and dl-PCBs by congeners. In this work, the LB value is employed to show which congeners are detected and what their contribution is.

**Figure 3:**
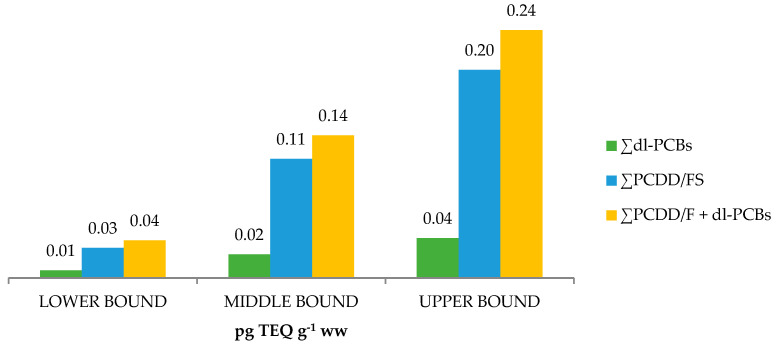
PCDD/Fs + dl-PCBs arithmetic mean levels in meconium.

The sum levels of the 17 PCDD/F congeners and 12 dl-PCBs congeners at the upper-bound (UB), expressed as picograms of toxic equivalency quantity (TEQ) per gram of wet weight (ww), in the 10 analysed samples ranged from 0.19 to 0.31 pg TEQ g^−1^ ww (see Table 3). The arithmetic means of the overall levels of the PCDD/Fs + dl-PCBs, expressed as TEQ, are shown in Figure 3 as UB, middle-bound (MB), and LB. Considering the obtained results, ∑PCDD/Fs contributed more than 75% to the total obtained levels.

Table 4 reflects all the described studies in the literature that analyse some of the congeners assessed in this study in meconium samples. The main limitation when comparing the results obtained is the expression, as there is no common ordering. The average mean of PCB-118 in Jeong et al., 2016 [16] and Veyhe et al., 2013 [15] were described in the same units, with the results obtained in this study (average of 27.4 pg g^−1^ ww) being higher than in Korea (1.66 pg g^−1^ ww) [16] and lower than in Norway (49 pg g^−1^ ww) [15]. The obtained results in other studies (Morokuma et al., 2017 [7], Alvarez-Silvares et al., 2021 [18], and T. Fernandez-Cruz et al., 2020 [2]) were expressed in pg g^−1^ lipid weight (lw), so they cannot be properly compared with the obtained results due to the low fat percentage obtained, as it could overestimate the concentration of the assessed compounds.

## 3. Materials and Methods

### 3.1. Subject Recruitment and Sample Collection

In total, 10 pregnant mothers were recruited between August and September 2022 at the Hospital General of Valencia (Spain). The mothers had signed an informed consent to participate.

There were several reasons for being rejected to participate in the study: if the mother had a multiple birth, if she had meconium amniotic fluid at the time of birth, or if there was a language barrier that prevented her from understanding what the project was about and what it implied.

The study was previously approved by the Ethical Committee of the General University Hospital of Valencia (Resolution 49/2022). The meconium samples were immediately collected after deposition and stored at −20 °C until transfer to the Biobank for the Biomedical and Public Health Research (IBSP-CV BioBank; PT13/0010/0064), and they were integrated into the Spanish National Biobank Network and the Valencian Biobanking Network, where they were stored at −80 °C until analysis. The data were processed in accordance with ethical and legal considerations at both national and EU levels, specifically adhering to the regulations set forth in the General Data Protection Regulation.

### 3.2. Standards and Reagents

Mass-labelled ^13^C_12_-PCDD/F and ^13^C_12_-dl-PCB stock solutions (1613EPA-LCS and WP-LCS) were employed as surrogates with concentrations of 100 and 1000 ng mL^−1^, respectively. These surrogates were purchased from Wellington Laboratories (Guelph, ON, Canada). A working solution of WP-LCS (50 ng mL^−1^) was prepared by diluting it with nonane. Additionally, mass-labelled ^13^C_12_-PCDD/F and ^13^C_12_-dl-PCB stock solutions (1613EPA-ISS and WP-ISS) were used as internal standards with concentrations of 200 and 1000 ng mL^−1^, respectively. These internal standards were also obtained from Wellington Laboratories (Guelph, ON, Canada) and diluted with nonane to create working solutions of 100 and 50 ng mL^−1^, respectively.

Commercial calibration standards solutions of PCDD/F (EPA 1613-CSL, EPA 1613-CS0.5, EPA 1613-CS1, EPA 1613-CS2, EPA 1613- CS3, EPA 1613-CS4, and EPA 1613-CS5) with 2,3,7,8-TCDD concentrations of 0.1, 0.25, 0.5, 2, 10, 40, and 200 ng mL^−1^, respectively, and calibration standard solutions of dl-PCBs (WP-CS1, WP-CS2, WP-CS3, WP-CS4, WP-CS5, WP-CS6, and WP-CS7) with concentrations of 0.1, 0.5, 2, 10, 40, 200, and 800 ng mL^−1^, respectively, were obtained from Wellington Laboratories (Canada).

Solvents (dichloromethane, n-hexane, toluene, nonane, and ethyl acetate) and PowerPrep consumables were purchased according to Lopez et al. (2021) [24].

### 3.3. Extraction Procedures

This study assessed three different extraction methodologies: ultrasonic-assisted extraction (UAE), selective pressurized liquid extraction (SPLE), and microwave-assisted extraction (MAE). To prepare the samples, 20 and 50 μL of the surrogate solutions (1613EPA-LCS and WP-LCS working solutions) were added to the samples, and they were allowed to interact for 30 min prior to extraction. For each procedure, these assays were performed in triplicate, using real meconium samples to perform the tests.

#### 3.3.1. Ultrasonic Assisted Extraction (UAE)

The sample was placed in a flask, and after the interaction time (30 min) with the working solutions, 30 mL of hexane:dichloromethane (1:1) was added and sonicated for 30 min at 40 °C in an ultrasound bath from JP Selecta (Spain). The supernatant was filtered and transferred to a Turbovap tube. Before filtration, anhydrous sodium sulphate was added to remove water.

#### 3.3.2. Selective Pressurized Liquid Extraction (SPLE)

Approximately 0.5 g of meconium sample was employed, then pulverized with 8 g of diatomaceous earth and placed into a 66 mL stainless steel extraction cell with a little anhydrous sodium sulphate (Sigma Aldrich, Steinheim, Germany) before being placed in the accelerated solvent extraction system (ASE 350, Dionex, Sunnyvale, CA, USA). The samples were subjected to extraction using n-hexane and dichloromethane (1:1) as a solvent. The extraction was performed at 100 °C under a pressure of 1500 psi for 5 min and three static cycles with a static time of 5 min. A flush volume equivalent to 90% of the extraction cell capacity was used. Following extraction, pressurized nitrogen was used to purge the extract for 100 s. The extract was then filtered and transferred to a Turbovap tube, with anhydrous sodium sulphate added beforehand to remove any water present.

#### 3.3.3. Microwave-Assisted Extraction (MAE)

The extraction was conducted with a Mars System microwave from CEM Corporation (Matthews, NC, USA) in 40 mL of toluene as a solvent extractor. The temperature programme was set as follows: initial temperature and hold 20 °C for 0 s; initial ramp to 100 °C at 20 °C per minute (1200 W); second hold 100 °C for 20 min (1200 W). The extract was filtered and transferred to a Turbovap tube. Before filtration, anhydrous sodium sulphate was added to remove water.

### 3.4. Clean Up

Two different fractions for the PCDD/Fs and dl-PCBs were obtained following the procedure described by Hernandez et al. (2020). Briefly, a purification process was carried out on a multicolumn setup (Power-PrepTM, Fluid Management Systems, Billerica, MA, USA) employing silica ABN, alumina, and carbon columns [10].

### 3.5. GC-HRMS Analysis

The analyses of the PCDD/Fs and dl-PCBs were performed by GC-HRMS on a Trace 1310 Gas Chromatograph (Thermo Scientific, Milan, Italy) equipped with a TG-Dioxin capillary column (60 m × 0.25 mm × 0.25 μm) and coupled to a DFS^TM^ High-Resolution Mass Spectrometer (Thermo Scientific, Bremen, Germany). The instrument used for the analysis had a set mass resolution of approximately 10,000 R. A split/splitless injector was utilized, injecting 2 μL for the PCDD/Fs and 1 μL for the dl-PCBs in the splitless mode. The injector temperature was set at 290 °C. The transfer line was maintained at 260 °C, and the ionization source was maintained at 260 °C. The identification of dioxins, furans, and dl-PCBs was carried out by comparing the ion ratios and retention times with their corresponding standards. The M and M + 2 ions were monitored for tetrachlorinated congeners, while the M + 2 and M + 4 ions were monitored for the other evaluated compounds.

For the analysis of the dioxins/furans, the GC temperature program can be found in another study carried out by our research group [24].

The assessment of the PCDD/F and dl-PCB congeners followed the EPA 1613 and EPA 1668B procedures, respectively. The quantification was achieved through isotopic dilution, and TargetQuan 4.0 software (Thermo Scientific, Milan, Italy) was employed.

### 3.6. Quality Assurance/Quality Control (QA/QC)

The current study adhered to the quality assurance system outlined by ISO/IEC/EN 17025 and the quality assurance requirements specified in Commission Regulation 2017/644 regarding the analysis of dioxin levels, dioxin-like PCBs, and non-dioxin-like PCBs [25].

The recoveries of each surrogate in every sample were assessed, and only those with recoveries ranging from 60% to 120% were considered valid. The recoveries outside of this range for individual congeners were acceptable if they contributed less than 10% to the total TEQ value derived from the sum of the PCDD/Fs and dioxin-like PCBs [25]. Furthermore, a reagent blank was included in each batch of samples to determine and adjust for any background interference from the laboratory, and the concentrations found in the reagent blank were subtracted from the actual sample results.

The determination of the limit of quantification (LOQ) for each congener followed the guidelines set out in the Guidance Document on the Estimation of the LOD and LOQ for Measurements in the Field of Contaminants in Feed and Food [26], in accordance with Commission Regulation 644/2017 [25]. The LOQ for each congener and sample was estimated based on the concentration of the analyte in the final extract of the sample, which produced a signal-to-noise ratio (S/N) of 3:1 at two different ions. Target Quan software 4.0, provided by Thermo Scientific, Milan, Italy, was used to calculate the LOQs.

## 4. Conclusions

A sensitive, selective, and reproducible methodology has been developed for PCDD/Fs and dl-PCBs in meconium. To our knowledge, this is the first study to jointly detect the 17 PCDD/Fs and 12 dl-PCBs cited in the Stockholm Convention in this human matrix by GC-HRMS.

Among the three compared extraction methodologies, it has been concluded that SPLE is the most suitable extraction technique due to the optimal ratio between the amount of extracted fat and the obtained recoveries for the labelled compounds.

The levels of PCDD/Fs ranged from n.d. to 1.93 pg g^−1^ ww (OCDD), and the dl-PCB concentrations oscillated from n.d. to 37.42 pg g^−1^ ww (PCB-118).

This analytical strategy can be applied in future biomonitoring programmes to assess prenatal exposure to PCDD/Fs and dl-PCBs.

## Figures and Tables

**Table 1:** Obtained results for PCDD/Fs in meconium samples (n = 10).

Congener	Detection Frequency (%)	Range (pg g^−1^ ww)	Average LOQ (pg g^−1^ ww)	AM ^a^ (pg g^−1^ ww)	AM ^a^ TEQ_2005_ (pg TEQ g^−1^ ww)			Main Congeners Contribution (%)
					UB	MB	LB	
2378-TCDF	80	n.d.–0.29	0.03	0.2	1.5 × 10^−2^	1.5 × 10^−2^	1.5 × 10^−2^	48.8
12378-PECDF	90	n.d.–0.35	0.05	0.3	7.0 × 10^−3^	7.0 × 10^−3^	6.9 × 10^−3^	28.3
23478-PECDF	10	n.d.–0.1	0.06	0.1	1.9 × 10 ^−2^	1.1 × 10 ^−2^	2.9 ×10^−3^	6.3
123478-HXCDF	0	n.d.	0.07	-	6.7 × 10^−3^	3.3 × 10^−3^	0.00	0.0
123678-HXCDF	0	n.d.	0.04	-	4.5 × 10^−3^	2.2 × 10^−3^	0.00	0.0
234678-HXCDF	0	n.d.	0.06	-	6.4 × 10^−3^	3.2 × 10^−3^	0.00	0.0
123789-HXCDF	0	n.d.	0.05	-	5.4 × 10^−3^	2.7 × 10^−3^	0.00	0.0
1234678-HPCDF	40	n.d.–0.29	0.03	0.2	8.6 × 10^−4^	8.0 × 10^−4^	7.4 × 10^−4^	3.0
1234789-HPCDF	0	n.d.	0.03	-	2.6 × 10^−4^	1.3 × 10^−4^	0.00	0.0
OCDF	0	n.d.	0.06	-	1.7 × 10^−5^	8.6 × 10^−6^	0.00	0.0
2378-TCDD	0	n.d.	0.04	-	3.8 × 10^−2^	1.9 × 10^−2^	0.00	0.0
12378-P × 10 CDD	0	n.d.	0.07	-	7.0 × 10^−2^	3.5 × 10^−2^	0.00	0.0
123478-HXCDD	0	n.d.	0.07	-	7.3 × 10^−3^	3.6 × 10^−3^	0.00	0.0
123678-HXCDD	0	n.d.	0.07	-	7.1 × 10^−3^	3.5 × 10^−3^	0.00	0.0
123789-HXCDD	0	n.d.	0.07	-	7.3 × 10^−3^	3.6 × 10^−3^	0.00	0.0
1234678-HPCDD	80	n.d.–0.57	0.07	0.4	3.3 × 10^−3^	3.2 × 10^−3^	3.1 × 10^−3^	12.4
OCDD	100	0.56–1.93	0.08	1.1	3.3 × 10^−4^	3.3 × 10^−4^	3.3 × 10^−4^	1.3

<LOQ: Less than the limit of quantification; n.d.: not detected. ^a^ Arithmetic mean.

**Table 2:** Obtained results for dl-PCBs in meconium samples (n = 10).

Congener	Detection Frequency (%)	Range (pg g^−1^ ww)	Average LOQ (pg g^−1^ ww)	AM ^a^ (pg g^−1^ ww)	AM ^a^ TEQ_2005_ (pg TEQ g^−1^ ww)			Main Congeners Contribution (%)
					UB	MB	LB	
PCB-81	90	n.d.–0.46	0.24	0.3	8.9 × 10^−5^	8.5 × 10^−5^	8.2 × 10^−5^	3.3
PCB-77	100	1.40–3.18	0.25	2.1	2.1 × 10^−4^	2.1 × 10^−4^	2.1 × 10^−4^	9.2
PCB-123	100	1.34–2.50	0.26	1.9	5.7 × 10^−5^	5.7 × 10^−5^	5.7 × 10^−5^	2.3
PCB-118	100	19.48–37.42	0.26	27.4	8.2 × 10^−4^	8.2 × 10^−4^	8.2 × 10^−4^	32.1
PCB-114	80	n.d.–2.02	0.26	1.2	3.1 × 10^−5^	3.1 × 10^−5^	3.0 × 10^−5^	1.0
PCB-105	100	7.52–11.82	0.27	9.4	2.8 × 10^−4^	2.8 × 10^−4^	2.8 × 10^−4^	11.3
PCB-126	10	n.d.–0.27	0.29	0.3	2.9 × 10^−2^	1.6 × 10^−2^	2.7 × 10^−3^	9.2
PCB-167	100	1.46–4.70	0.20	3.1	9.4 × 10^−5^	9.4 × 10^−5^	9.4 × 10^−5^	3.3
PCB-157	90	n.d.–4.18	0.21	1.8	5.0 × 10^−5^	5.0 × 10^−5^	4.9 × 10^−5^	1.6
PCB-156	100	3.79–22.62	0.21	9.2	2.8 × 10^−4^	2.8 × 10^−4^	2.8 × 10^−4^	9.5
PCB-169	20	n.d.–0.49	0.22	0.4	7.5 × 10^−3^	5.1 × 10^−3^	2.6 × 10^−3^	17.1
PCB-189	30	n.d.–3.90	0.88	2.1	4.4 × 10^−5^	3.1 × 10^−5^	1.9 × 10^−5^	0.2

n.d.: not detected. ^a^ Arithmetic mean.

**Table 3:** PCDD/Fs and dl-PCBs levels for each sample in pg TEQ g^−1^ ww.

Sample	UB			MB			LB		
	∑PCDD/Fs	∑dl-PCBs	∑(PCDD/F + dl-PCBs)	∑PCDD/Fs	∑dl-PCBs	∑(PCDD/F + dl-PCBs)	∑PCDD/Fs	∑dl-PCBs	∑(PCDD/F + dl-PCBs)
S1	0.172	0.043	0.215	0.095	0.030	0.125	0.018	0.016	0.034
S2	0.260	0.041	0.301	0.153	0.022	0.174	0.046	0.002	0.048
S3	0.209	0.034	0.243	0.119	0.018	0.137	0.029	0.002	0.031
S4	0.182	0.026	0.208	0.103	0.014	0.116	0.023	0.001	0.024
S5	0.241	0.052	0.293	0.139	0.033	0.173	0.038	0.014	0.052
S6	0.185	0.037	0.222	0.107	0.033	0.140	0.028	0.029	0.058
S7	0.266	0.042	0.308	0.140	0.022	0.162	0.014	0.002	0.016
S8	0.171	0.035	0.206	0.099	0.018	0.117	0.026	0.002	0.028
S9	0.160	0.029	0.189	0.090	0.015	0.106	0.021	0.002	0.023
S10	0.146	0.042	0.188	0.096	0.022	0.118	0.045	0.002	0.047

**Table 4:** Previous studies about the determination of POPs in meconium samples.

POPs	Mean	Common Compounds	Region	Reference
PCDD/Fs + dl-PCBs	2.9 ^a^	PCDD/Fs, PCB 77, 81, 126, 169	Japan (Fukuoka)	[7]
dl-PCBs	0.027 ^b^	PCB 77, 81, 105, 114, 118, 123, 126, 156, 157, 167, 169, 189	Spain (Ourense)	[18]
	0.29 ^b^	PCB 77, 81, 105, 114, 118, 123, 126, 156, 157, 167, 169, 189	Spain (Ourense)	[2]
	1.66 ^c^	PCB 118	Korea (Seoul, Anyang, Ansan, Jeju)	[16]
	49 ^c^	PCB 118	Norway (Nordland, Troms, Finnmark)	[15]
	0.67 ^d^	PCB 77, 81, 105, 114, 118, 123, 126, 156, 157, 167, 169, 189	China (Zhejiang)	[17]

^a^: pg TEQ g^−1^ lw. ^b^: ng g^−1^ lw. ^c^: pg g^−1^ ww. ^d^: pg TEQ g^−1^ dw.

## Data Availability

Not applicable.

## Supplementary Materials

The following supporting information can be downloaded at https://www.mdpi.com/article/10.3390/molecules28135006/s1, Table S1: Overview of extraction and clean-up methods in the analysis of PCDD/Fs and dl-PCBs in meconium.
