# Supplementary material for: Development of a Methodology for Determination of Dioxins and Dioxin-like PCBs in Meconium by Gas Chromatography Coupled to High-Resolution Mass Spectrometry (GC-HRMS)
Source: Molecules. 2023 Jun 26;28(13):5006. doi: 10.3390/molecules28135006 (PMC10343527; doi:10.3390/molecules28135006)
Supplement: Supplementary file 1 [file molecules-28-05006-s001.zip › molecules-2448740-supplementary.pdf]

Article

Supplementary Materials: Development of a methodology for determination of dioxins and dioxin-like PCBs in meconium by gas chromatography coupled to high-resolution mass spectrometry (GC-HRMS)

Iñaki Lacomba [1,2], Antonio López [1], Raquel Hervàs-Ayala [3] and Clara Coscollà [1,*]

[1] Foundation for the Promotion of Health and Biomedical Research of the Valencian Region, FISABIO-Public Health, Av. Catalunya, 21, 46020 Valencia, Spain
[2] Department of Analytical Chemistry, Univeristy of Valencia, Doctor Moliner 50, 46100 Burjassot, Spain
[3] Department of Obstetrics and Gynaecology, General University Hospital of Valencia, Valencia, Spain
* Correspondence: coscolla_cla@gva.es; Tel.: +34961926333

Table of Contents

Type	Captions	Page
Table S1	Overview of extraction and clean-up methods in the analysis of PCDD/Fs and dl-PCBs in meconium.	S3

Table S1. Overview of extraction and clean-up methods in the analysis of PCDD/Fs and dl-PCBs in meconium.

References	Country	Extraction method	Clean-up	Sample amount (g)	Analysis Technique	Common Compounds
Fernandez-Cruz et al., 2020	Spain	SPLE (ACN[a])	Dual-layer SPE EZ-POP cartridges	0.5	GC-QqQ-MS/MS	PCB 77, 81, 105, 114, 118, 123, 126, 156, 157, 167, 169, 189
Morokuma et al., 2017	Japan	Not specified	Not specified	5	HRGC-MS	7 PCDD, 10 PCDF, 4 PCB
Jeong et al., 2016	Korea	Soxhlet extraction (DCM[b]:HX[c] (3:1))	Multi-layered SPE containing anhydrous Na2SO4, silica, 22% (w/w) H2SO4-silica gel, 44% (w/w) H2SO4-silica gel, and 2% (w/w) KOH-silica gel	0.5-1.5	HRGC-HRMS	PCB 118
Veyhe et al., 2013	Norway	Solid-Liquid extraction (H2SO4 + HX[c]) vortex and ultrasonic	SPE automated cleaning using Florisil	0.5-0.7	GC-MS	PCB 118, 156
Álvarez-Silvares et al., 2021	Spain	SPLE (ACN[a])	Dual-layer SPE EZ-POP cartridges	0.5	GC-QqQ-MS/MS	PCB 118, 77, 126, 156, 157, 81, 105, 114, 167, 169, 189

[a]ACN: acetonitrile

[b]DCM: dichloromethane

[c]HX: hexane
